# Exploring the diversity of cannabis cannabinoid and non-cannabinoid compounds and their roles in Alzheimer's disease: A review

**DOI:** 10.1016/j.ibneur.2024.12.011

**Published:** 2024-12-20

**Authors:** Hanane Doumar, Hicham El Mostafi, Aboubaker Elhessni, Mohamed Ebn Touhami, Abdelhalem Mesfioui

**Affiliations:** a Laboratory of Biology and Health, Department of Biology, Faculty of Sciences, Ibn Tofail University, Kenitra, Morocco; b Laboratory of Materials Engineering and Environment: Modeling and Application, Department of Chemistry, Faculty of Sciences, Ibn Tofail University, Kenitra, Morocco

**Keywords:** Cannabis sativa, Alzheimer's disease, Neuroprotection, Cannabinoids, Multi-target therapy

## Abstract

Cannabis sativa is recognized for its chemical diversity and therapeutic potential, particularly in addressing neurodegenerative diseases such as Alzheimer's disease (AD). Given the complexity of AD, where single-target therapies often prove inadequate, a multi-target approach utilizing cannabis-derived compounds may offer promising alternatives. This review first highlights the chemical diversity of cannabis by categorizing its compounds into cannabinoids and non-cannabinoids. It then examines studies investigating the effects of these compounds on AD-related pathological features. By synthesizing existing knowledge, identifying research gaps, and facilitating comparative analysis, this review aims to advance future research and understanding. It underscores cannabis's potential as a multi-target therapeutic strategy for AD, contributing valuable insights to ongoing scientific discussions.

## Highlights

• Cannabis sativa exhibits rich chemical diversity, categorized into cannabinoid and non-cannabinoid compounds.
• The plant’s therapeutic potential is particularly relevant for treating neurodegenerative diseases like AD.
• The discovery of the endocannabinoid system underscores the importance of cannabis-derived compounds in AD research.
• A multi-target approach with cannabis compounds may overcome the limitations of single-target therapies in AD.
• The review synthesizes existing research, identifies gaps, and aims to improve future studies on cannabis and AD.

## 1 Introduction

Alzheimer's disease (AD) is the most common form of dementia, representing a progressive and irreversible neurological disorder characterized by declining memory, cognition, and behavior (WHO, 2023). It affects over 55 million people worldwide, with a significant prevalence in low- and middle-income countries. The World Health Organization (WHO) estimates that the number of individuals living with dementia could rise to 139 million by 2050, alongside a doubling of the financial burden, from $1.3 trillion in 2019 to $2.8 trillion by 2030. AD profoundly impacts individuals, families, and society as a whole (Alzheimer’s Disease International, 2023).

(Zhang and Gordon, 2018; Birks and Grimley Evans, 2015)The scientific and pharmaceutical research community has faced significant challenges in developing effective treatments for AD due to the complexity of the human brain (Abubakar et al., 2022). After nearly two decades of intensive pharmacologic research, current treatments include cholinesterase inhibitors like tacrine (Sameem et al., 2017), donepezil (Zhang and Gordon, 2018), rivastigmine (Birks and Grimley Evans, 2015) and galantamine (Nakayama et al., 2017), which temporarily improve memory and cognitive function, along with memantine for moderate to severe cases. However, these therapies primarily address symptoms rather than the underlying disease mechanisms (Husna Ibrahim et al., 2020).

The reasons for the high failure rate in AD treatment trials are complex and cannot be attributed to a single cause. For example, in 2018, more than 50 % of drugs in Phase III trials focused on targeting beta-amyloid (Aβ), but by 2024, this focus had diminished to only 22 %, highlighting the challenges associated with amyloid-targeted therapies (Cummings et al., 2018, Cummings et al., 2024). Among the notable therapeutic strategies are monoclonal antibodies aimed at reducing amyloid burden, which are considered disease-modifying, even though they are not etiological treatments (Budd Haeberlein et al., 2022). Recent approvals include aducanumab and lecanemab, with donanemab currently under review (CH et al., 2023; Budd Haeberlein et al., 2022; Sims et al., 2023; FDA, 2024). Despite their promise, these treatments are effective mainly in the early stages of the disease and may have side effects such as swelling or microhemorrhages, in addition to high treatment costs (Reardon, 2023, Wu et al., 2023).

Indeed, complex neurological pathologies like Alzheimer's are unlikely to be effectively addressed with a single-target solution. A more holistic approach may prove more efficient. In this context, it may be beneficial to return to nature, which has consistently offered effective treatments for numerous human diseases. This is evidenced by the fact that over 60 % of drugs approved between 1981 and 2019 are derived from or inspired by natural compounds. Natural products play a critical role in drug discovery, with 64.9 % of small molecule drugs approved for cancer treatment since 1981 being based on natural product structures (Newman and Cragg, 2020).

Cannabis is a natural plant with a long history of human use, particularly in therapeutics. Historically, cannabis was regarded as a neurotoxic and addictive natural product with significant risks. However, recent research has sparked renewed interest in its medicinal benefits (Crocq, 2020). This shift in perception is largely due to the discovery of phytocannabinoids, which interact with cannabinoid receptors to modulate biological responses such as inflammation and pain (Crocq, 2020). Studies are now focusing on the therapeutic properties of various chemical compounds extracted from cannabis, emphasizing the importance of appropriate extraction methods and dosages. Cannabis is notable for its remarkable chemical diversity, containing over 550 bioactive compounds with promising therapeutic potential (Rock and Parker, 2021). This is further supported by research highlighting not only cannabinoids like CBD and THC but also non-cannabinoid compounds, including terpenes and flavonoids, which have shown potential in treating neurodegenerative disorders (Laaboudi et al., 2024; Yadav et al., 2023). Laaboudi et al. (2024), further emphasize the role of secondary metabolites, such as terpenes and phenolic compounds, in enhancing the therapeutic effects of cannabinoids through the "entourage effect." These non-cannabinoid compounds play a vital role in modulating inflammation, oxidative stress, and other key pathological processes, which are crucial in the treatment of Alzheimer's disease. Yadav et al. (2023), expand on this, noting that cannabis contains a wide array of compounds beyond cannabinoids, including over 150 terpenes and 42 phenolic compounds, each with distinct pharmacological activities such as anti-inflammatory, anti-cancer, and neuroprotective effects.

In addition, Tyrakis et al. (Tyrakis et al., 2024) extensively review the endocannabinoid system, focusing on its role in Alzheimer’s disease. The article examines the modulation of the system’s pathways and how various cannabinoids, including non-selective cannabinoid agonists, impact key pathological features of Alzheimer's, such as neurodegeneration and inflammation. Their synthesis of studies from 2014 to 2024 identifies significant mechanisms through which cannabinoids improve memory, cognition, and behavioral symptoms in Alzheimer's disease models. Although the role of phytocannabinoids is only briefly addressed in the article, the findings align with growing evidence that modulating the endocannabinoid system can provide therapeutic benefits for Alzheimer’s patients.

While the referenced studies provide valuable insights into the broader pharmacological properties of cannabis, our review not only catalogs over 323 cannabis-derived chemical compounds, emphasizing both well-studied cannabinoids and often-overlooked non-cannabinoid compounds, but also specifically examines their effects on AD-related pathological features. We highlight the synergistic effects of cannabis extracts, including flavonoids and terpenes, through the entourage effect, offering a more comprehensive understanding of cannabis's therapeutic potential in Alzheimer's treatment. Additionally, this review underscores the growing body of clinical and preclinical studies, aiming to fill gaps in knowledge regarding the most effective extraction methods, dosages, and synergistic effects of cannabis compounds in Alzheimer's treatment, an area not fully explored in the existing literature.

### 1.1 Classification of Cannabis components

As previously mentioned, cannabis is a chemically diverse plant with over 550 identified compounds, including more than 100 phytocannabinoids such as Δ9-tetrahydrocannabinol (Δ9-THC) and cannabidiol (CBD) (Rock and Parker, 2021). This diversity has significantly expanded due to advances in extraction and separation technologies, and the rapidly growing cannabis industry underscoring the importance of product development and extraction methods (Alves et al., 2021, Brighenti et al., 2024).

To classify cannabis compounds, several criteria can be used, including chemical structure, biosynthetic pathway, pharmacological activity, functional groups, plant part origin, extraction method, and therapeutic potential. In this review, we categorize cannabis compounds into two main groups based on their chemical structure and biological activity: cannabinoids and non-cannabinoids. Each category, sub-categories and its compounds types are detailed in Table 1, Table 2, following a previous study (Radwan et al., 2021).

**Table 1:** Chemical Composition of Phytocannabinoids Extracted from Cannabis: Sub-categories, Compounds, Extraction Methods.

**Phytocannabinoids Sub-categories**	**Compounds**	**Extraction methods**	**References**
cannabichromene (CBC), (Nine Compounds)	Cannabichromene (CBC)	Isolated from the hexane extract of hashish, using column chromatography (florisil column) in 1966	(Gaoni and Mechoulam, 1966)
	cannabichromenic acid (CBCA)	Isolated from the benzene extract of hemp, using silica-gel column chromatography	(Shoyama et al., 1975)
	Cannabivarichromene ( ± CBCV)	Identified through GC-MS analysis.	
	annabichromevarin (+ CBCV)	Isolated from a benzene extract of the “Meao” variant of cannabis from Thailand	
	cannabichromevarinic acid (CBCVA)		
	( ± )−4-acetoxycannabichromene	Isolated from high potency *C. sativa* using silica-gel VLC, normal-phase silica HPLC, and reverse-phase silica (C_18_) HPLC.	(Radwan et al., 2009)
	( ± )−3”-hydroxy-Δ^4”^-cannabichromene		
	(–)−7-hydroxycannabichromane		
	CBC-C_3_ derivative	Reported, after using spectral analysis to be 2-methyl−2-(4-methyl−2-pentyl)−7-propyl-2H−1-benzopyran−5-ol	(Morita M and Ando H, 1984)
Cannabidiol (CBD), (10 Cannabinoids)	CBD,	Isolated from an ethanolic extract (red oil) of Minnesota wild hemp. It was purified as a *bis*−3,5-dinitrobenzoate crystalline derivative	(Adams et al., 1940)
	CBDA-C_5_	Isolated from the fresh tops and leaves of *C. sativa* after extraction with benzene and identified by comparing its UV spectrum with that of CBD derivatives	(Krejci Z. and Santavy F, 1955)
	Cannabidiol monomethylether (CBDM-C_5)_	Isolated from the decarboxylated ethanol extract of hemp leaves on using florisil and silica gel column chromatography. CBDM	(Shoyama et al., 1972)
	CBD-C_4_	Obtained from the ethyl acetate extract of cannabis resin and leaves after derivatization	(D. I. Harvey, 2011)
	Cannabidivarin (CBDV-C_3_)	Reported from hashish through silica gel chromatography	(Vollner et al., 1969)
	CBDVA	Isolated from the benzene extract of Thai cannabis, which was chromatographed on a polyamide column eluted with H_2_O: MeOH (1:1–1:6).	(Shoyama et al., 1977)
	Cannabidiorcol (CBD-C_1_)	Identified in the hexane extract of Lebanese hashish by combined gas chromatography–mass spectrometry [	(Vree et al., 2011)
	cannabidihexol (CBDH)	Purified using a semi-preparative C18 HPLC using a mixture of ACN/0.1 aqueous formic acid as a mobile phase.	(Citti et al., 2019)
	cannabidiphorol (CBDP)		
	Cannabitwinol, (CBDD)	isolated from the hexane extract of hemp The hexane extract was chromatographed on a Sigel column, which was eluted with hexane/CH_2_Cl_2_ followed by semi preparative C18-HPLC using a mixture of ACN/H_2_O/formic acid (7:3:0.1) as the mobile phase	(Chianese et al., 2020)
cannabielsoin (CBE), (Five Compounds)	cannabielsoin (CBE-C5)	Detected in the ethanolic extract of Lebanese hashish. The extract was subjected to counter current distribution followed by GCMS analysis	(Bercht et al., 1973)
	cannabielsoin acid A (CBEAA)	Isolated from hashish; the structural elucidation was carried by NMR spectroscopy and chemical transformations	(Shani and Mechoulam, 1974)
	cannabielsoin acid B (CBEAB)		
	Cannabielsoin-C3 (CBE-C3)	Reported from cannabis in 1978	(ElSohly and Slade, 2005; Grote and Spiteller, 1978)
	Cannabielsoic acid B-C3 (CBEAB-C3)		
cannabigerol (CBG), (16 Cannabinoids)	cannabigerol ((*E*)-CBG	Isolated from cannabis resin, using florisil chromatography	(Gaoni and Mechoulam, 1964)
	Cannabigerolic acid (CBGAA)		(Shoyama et al., 1975)
	monomethyl ether of CBGAA (CBGAM)		
	monomethyl ether of (*E*)-CBG (CBGM)	Isolated from a benzene extract of hemp by heating the extract with toluene for seven hours and purifying using column chromatography (silica-gel column) with benzene as the eluent	(Obata and Ishikawa, 1966)
	Cannabigerovarin (CBGV)	Isolated from a benzene extract of “Meao variant” cannabis from Thailand	(Shoyama et al., 1975)
	cannabigerovarinic acid (CBGVA)		
	cannabinerolic acid ((*Z*)CBGA)	Isolated from an acetone extract of the leaves of a Mexican strain of *C. sativa*, using silica-gel column chromatography.	(Taura et al., 1995)
	5-acetyl−4-hydroxy-cannabigerol	Isolated from the buds of high potency *C. sativa* using normal phase HPLC of the polar fractions	(Radwan et al., 2009)
	( ± )−6,7-*trans*-epoxycannabigerolic acid	isolated from high potency *C. sativa* (grown in Mississippi) by the application of various chromatographic techniques (VLC, flash chromatography, and HPLC)	(Radwan, Ross, et al., 2008)
	( ± )−6,7-*cis*-epoxycannabigerolic acid		
	( ± )−6,7-*cis*-epoxycannabigerol		
	( ± )−6,7-*trans*-epxoycannabigerol (		
	camagerol	Isolated from the aerial parts of a *C. sativa* strain, Carma, using reverse-phase (C_18_) silica-gel column chromatography, followed by normal-phase silica gel column chromatography and, finally, normal phase (NP)-HPLC. The wax of the aerial parts of the Carma strain was hydrolyzed and purified, using silica and alumina column chromatography, resulting in waxy and non-waxy fractions.	(Appendino et al., 2008)
	farnesyl prenylogue of cannabigerol (sesquicannabigerol	Isolated from one of the waxy fractions	
cannabicyclol (CBL), Three3 Compounds	CBL	Isolated from hashish by thin layer chromatography	(Korte and Sieper, 1964, Mechoulam and Gaoni, 1967)
	Cannabicyclolic acid (CBLA)	Obtained from the benzene extract of cannabis. The benzene extract was chromatographed on a polyamide column using methanol water as a mobile phase. CBLA was isolated as a methylated derivative and considered to be an artifact formed when CBCA is naturally irradiated during storage	(Mechoulam and Gaoni, 1967)
	Cannabicyclovarin (CBLV)	Detected in the ether extract of Congo marihuana and was identified by GLC and GCMS	(Vree et al., 1972)
	cannabichromenic acid (CBCA,)	Isolated from the high potency variety of *C. sativa* and chemically identified based on NMR and high-resolution mass (HR-MS) analysis in 2009	(Radwan et al., 2009)
cannabinol (CBN), (11 Compounds)	Both 8-hydroxycannabinol		
	8-hydroxy cannabinolic acid A		
	1′*S*-hydroxy-cannabinol	Isolated from the same cannabis variety (high potency *C. sativa*), and their chemical structures were confirmed by GC-MS analysis	(Ahmed et al., 2015)
	4-terpenyl cannabinolate		
	CBN-C5	Isolated from the high potency variety of *C. sativa* since 1980	(Turner et al., 1980)
	CBNA-C5		
	CBN-C4		
	CBN-C3		
	CBN-C2		
	CBN-C1		
	CBNL-C5		
cannabinodiol (CBND) (Two Cannabinoids)	cannabinodivirin (CBND-C_3_)	Detected in hashish by GC-MS analysis	(Van Ginneken C. et al., 1972)
	(CBND-C_5_)		
cannabitriol (CBT), (9 Compounds)	Nine CBT-type cannabinoids, including (−)-*trans*-CBT-C_5_	isolated from cannabis.	(Obata and Ishikawa, 1966)
	(+)-*trans*-CBT-C_5_	Isolated by ElSohly et al. from the ethanolic extract of cannabis, which was chromatographed on a silica gel column and identified by GCMS	(M A Elsohly et al., 1977)
	( ± )-*cis*-CBT-C_5_		
	( ± )-*trans*-CBT-C_3_		
	CBT-C_3_-homologue		
	(−)-*trans*-CBT-OEt-C_5_		
	(–)-*trans*-CBT-OEt-C_3_		
	8,9-Di-OH-CBT-C_5_		
	CBDA-C_5_ 9-OH-CBT-C_5_ ester		
(−)-Δ^8^-*trans*-tetrahydrocannabinol (Δ8-THC), (5 Cannabinoids)	Δ^8^-THC,	Isolated in 1966 from the leaves and flowers of Cannabis grown in Maryland. Δ^8^-THC was purified from the petroleum ether extract through silicic acid column chromatography using benzene and an eluent	(Hively et al., 1966)
	Δ^8^-THCA	Isolated as the methyl ester from a Cannabis plant of Czechoslovakian origin	(Krejcí and and Šantavý, 1975)
	10α-hydroxy-Δ^8^-tetra-hydrocannabinol	Isolated from high-potency *C. sativa* using 1D and ^2^D NMR spectra	(Ahmed et al., 2015, Radwan et al., 2015)
	10β-hydroxy-Δ^8^-tetra-hydrocannabinol		
	10a-α-hydroxy−10-oxo-Δ^8^-tetrahydrocannabinol		
(−)-Δ^9^-*trans*-tetrahydrocannabinol (Δ^9^-THC), (25 cannabinoids)	Δ^9^-THC,	Found in hexane extract of hashish using column chromatography over florisil followed by alumina efficient, preparative C_18_ HPLC method was developed for the purification of Δ^9^-THC from the distillate	(Gaoni and Mechoulam, 1964)
	Δ^9^-THCAA	Cellulose powder column (eluted with a mixture of hexane and dimethylformamide) followed by preparative thin layer chromatography The isolation of Δ^9^-THCAA was also Reported using an acid–base extraction procedure	(Yamauchi et al., 1967)
	Δ^9^-THCAB	Reported from a hashish sole, using a silicic acid column eluted with a mixture of diethyl ether in petroleum ether.	(Rosenqvist et al., 1975)
	(−)-Δ^9^-*trans*-tetrahydrocannabinol-C_4_ (Δ^9^-THC-C_4_)	GC-MS	(Harvey, 2011)
	-Δ^9^-*trans*-tetrahydrocannabinolic acid A-C_4_ (Δ^9^-THCAA-C_4_)	GC-MS	
	(−)-Δ9-trans-tetrahydrocannabivarin (Δ9-THCV,)	Isolated from a cannabis tincture of Pakistani origin, using the counter-current distribution technique to isolate the compound from a light petroleum ether extract	(Gill, 1971)
	(−)-Δ9-trans-tetrahydrocannabivarinic acid (Δ9-THCVAA)	In 1973, the isolation of (−)-Δ9-trans-tetrahydrocannabivarinic acid (Δ9-THCVAA) from fresh *Cannabis sativa* leaves from South Africa was reported. The methyl ester of this cannabinoid produced a characteristic fragmentation pattern that was 28 mass units less.	(Shoyama et al., 1977)
	(−)-Δ^9^-*trans*-tetrahydrocannabiorcol (Δ^9^-THCO or Δ^9^-THC_1_,	A light petroleum ether extract was prepared from Brazilian *Cannabis sativa* (marijuana) to isolate non-polar cannabinoids and lipid-soluble compounds	(Turner et al., 1973)
	(−)-Δ^9^-*trans*-tetrahydrocannabiorcolic acid (Δ^9^-THCOAA,)	GC-MS analysis of different cannabis samples	(Harvey, 2011)
	(−)-Δ^9^-*trans*-tetrahydrocannabinal (Δ^9^-THC aldehyde)	Isolated from a high potency variety of *C. sativa* by applying VLC (Vacuum Liquid Chromatography), silica gel column chromatography, and HPLC	(Ahmed et al., 2015)
	(−)-Δ^9^-*trans*-tetrahydrocannabinolate)	Isolated from the hexane extract of the same high potency variety of *C. sativa* using various chromatographic techniques, such as vacuum liquid chromatography (VLC), C_18_ semi-preparative HPLC, and chiral HPLC.	(Ahmed, Ross, Slade, Radwan, Zulfiqar, et al., 2008)
	α-fenchyl (−)-Δ^9^-*trans*-tetrahydrocannabinolate		
	*epi*-bornyl (−)-Δ^9^-*trans*-tetrahydrocannabinolate		
	bornyl (−)-Δ^9^-*trans*-tetrahydrocannabinolate		
	α-terpenyl (−)-Δ^9^-*trans*-tetrahydrocannabinolate		
	4-terpenyl (−)-Δ^9^-*trans*-tetrahydrocannabinolate		
	α-cadinyl (−)-Δ^9^-*trans*-tetrahydrocannabinolate		
	γ-eudesmyl (−)-Δ^9^-*trans*-tetrahydrocannabinolate	Spectroscopic analysis, including ¹H NMR, ¹ ³C NMR, and ^2^D NMR, alongside GC-MS analysis, were employed to characterize the isolated compounds.	
	8α-hydroxy-(−)-Δ^9^-*trans*-tetrahydrocannabinol	High-potency *C. sativa* was processed using multiple chromatographic techniques, including silica gel VLC, C18-solid phase extraction (SPE), and HPLC.	(Radwan et al., 2015)
	8*β*-hydroxy-(−)-Δ^9^-*trans*-tetrahydro cannabinol		
	11-acetoxy- (−)-Δ^9^-*trans*-tetrahydrocannabinolic acid A		
	8-oxo-(−)-Δ^9^-*trans-*tetrahydrocannabinol		
	Cannabisol	High CBG content using flash silica gel chromatography eluted with hexane/CHCl_3_ (1:1)	(Zulfiqar et al., 2012)
	(−)-Δ^9^-*trans*-tetrahydrocannabiphorol	Recently isolated from the hexane extract of *C. sativa* inflorescences of an Italian origin (strain CIN-RO). The hexane extract was cooled at −20 °C for 48 h to remove waxes by precipitation. The dewaxed extract was subjected to semi-preparative liquid chromatography on a C18 stationary phase column to isolate compounds 24 and 25 after heating the corresponding acids at 120 °C for 2 h as clear oil.	(Citti et al., 2019, Linciano et al., 2020)
	(−)-Δ^9^-*trans*-tetrahydrocannabihexol		
miscellaneous-type cannabinoids (30 Compounds)	dehydrocannabifuran (DCBF-C5)	The cyclohexane-methanol extract of Afghan hashish with micropreparative GC and TLC	(Friedrich-Fiechtl and Spiteller, 1975)
	cannabifuran (CBF-C_5_)		
	8-hydroxy-isohexahydrocannabivirin (OH-iso-HHCV-C_3_)		
	10-oxo-Δ^6a(10a)^-tetrahydro-cannabinol (OTHC)		
	cannabicitran		
	(–)-Δ^9^-*cis*-(6a*S*,10a*R*)-tetrahydro-cannabinol (*cis*-Δ^9^-THC)	from a petroleum extract of marihuana by Smith and Kampfert in 1977. The extract was purified on a florsil column followed by preparative TLC [	(Smith and Kempfert, 1977)
	cannabicoumaronone (CBCON-C_5_)	Isolated from a South African Cannabis variant after hexane extraction and chromatography on silica and polyamide columns. Its chemical structure was determined by spectral means (IR, GCMS, UV,^1^H NMR) and by synthesis	(Boeren et al., 1977)
	cannabiripsol (CBR)		
	cannabitetrol (CBTT)	Isolated from an ethanolic extract of Lebanese hashish. It was purified by counter-current distribution and silica gel chromatography. Its chemical structure was determined by GCMS, IR and^1^H NMR analyses	(Bercht and Paris, 1974)
	cannabichromanone-C_5_ (CBCN-C_5_)	Isolated from cyclohexane-methanol extract of Afghan hashish	(Friedrich-Fiechtl and Spiteller, 1975)
	cannabichromanone-C_3_ (CBCN-C_3_)	ElSohly and Slade reported the details of the isolation chemical identification of	(ElSohly and Slade, 2005)
	( ± )-Δ^7^-*cis*-isotetrahydrocannabivarin-C_3_ (*cis*-iso-Δ^7^-THCV,)		
	(–)-Δ^7^-*trans*-(1 *R*,3 *R*,6 *R*)-isotetrahydrocannabi- varin-C_3_ (*trans*-iso-Δ^7^-THCV,)		
	(–)-Δ^7^-*trans*-(1 *R*,3 *R*,6 *R*)-isotetrahydrocannabinol-C_5_ (*trans*-iso-Δ^7^-THC,)		
	cannabichromanone B	Isolation of these compounds was performed using semi-preparative C_18_ HPLC	(Ahmed, Ross, Slade, Radwan, Khan, et al., 2008)
	cannabichromanone C		
	cannabichromanone D		
	(–)-(7 *R*)-cannabicoumarononic acid	Isolated from the buds and leaves of the same variety of cannabis (high potency *C. sativa*) using several chromatographic techniques, including silica-gel VLC, solid-phase extraction, reverse-phase columns (C_18_ SPE), and normal-phase HPLC	(Radwan et al., 2008)
	4-acetoxy−2-geranyl−5-hydroxy−3-*n*-pentylphenol		
	2-geranyl−5-hydroxy−3-*n*-pentyl−1,4-benzoquinone		
	5-acetoxy−6-geranyl−3-*n*-pentyl−1,4-benzoquinone	Isolated on silica gel column chromatography followed by normal-phase HPLC	
	cannabimovone (CBM)	Isolated from a non-psychotropic variety of *C. sativa* from a polar fraction of hemp by using flash chromatography over reverse-phase C_18_ silica gel followed by normal-phase HPLC. The chemical identity of CBM (117) was revealed by a combination of 1D and ^2^D NMR along with ESI-MS spectroscopic techniques]	(Taglialatela-Scafati et al., 2010)
	cannabioxepane, (CBX)	Isolated in 2011 from a cannabis variety called Carmagnola by applying many chromatographic techniques including RP−18 column, silica gel column chromatography, and NP-HPLC chromatography	(Taglialatela-Scafati et al., 2010)
	0*α*-hydroxy-Δ^9,11^-hexahydrocannabinol	Isolated from a high potency variety of *C. sativa* and chemically elucidated by 1D and ^2^D NMR and HRMS analyses	(Ahmed et al., 2015, Radwan et al., 2015)
	9*β*,10*β*-epoxyhexahydrocannabinol		
	9*α*-hydroxyhexahydrocannabinol		
	7-oxo−9*α*-hydroxyhexa-hydrocannabinol,		
	10*α*-hydroxyhexahydrocannabinol		
	10a*R*-hydroxyhexahydrocannabinol		
	9*α*-hydroxy−10-oxo-Δ^6a,10a^-THC

**Table 2:** Chemical Composition of Non-Cannabinoid Compounds Extracted from Cannabis: Sub-categories, Compounds, Extraction Methods. Terpenes.

Non-Cannabinoids Sub-categories		Compounds	Extraction method	References
Non-cannabinoid phenols (42 compounds)	Spiro-indans (16 compounds)	Cannabispiran	Isolated In 1976 From An Indian Cannabis Variety Using Silica Gel Column Chromatography	(Ottersen et al., 1976)
		Cannabispirone; Cannabispirenone	Also Identified From The South African Cannabis Variety	(Bercht et al., 1976)
		Cannabispirenone Isomer	Was isolated with interchangeable methoxy and hydroxyl groups, from Mexican marihuana, and its chemical structure was established by^1^H NMR and EIMS analysis,	(Kettenes-van den Bosch and Salemink, 1978)
		Cannabispiradienone	Isolated From Thai Cannabis, And Its Chemical Structure Was Elucidated Based On^1^h Nmr Spectroscopy And Confirmed By Hydrogenation To Give Cannabispiran (126)	(Cromble et al., 1979)
		Cannabispirol	Detected by Yukihiro and Nishioka in the benzene extract of the dried leaves of Japanese cannabis. The benzene extract was chromatographed on a polyamide column followed by silica gel chromatography to yield compounds 130 and 131	(Kettenes-van den Bosch and Salemink, 1978)
		Acetyl Cannabispirol		
		5-hydroxy−7-methoxyindan−1-spiro-cyclohexane	Isolated From An Ethanolic Extract Of A Seized Hashish Sample From Saudi Arabia;The Methanolic Fraction Of Hashish Was Subjected To Flash Chromatography and further purified through silica gel column chromatography to afford this 3 compounds	(El-Feraly et al., 1986)
		7-hydroxy−5-methoxyindan−1-spiro-cyclohexane		
		5,7-dihydroxyindan−1-spiro-cyclohexane		
		Isocannabispiran	Isolated from a panamanian variety of cannabis by repeated chromatography. The structure was chemically elucidated as 5′-hydroxy−7′-methoxy-spiro-(cyclohexane−1,1′-indan)−4-one by spectroscopic means as well as direct comparison with cannabispiran	(H. N. ElSohly and Turner, 1982)
		7-O-methyl-cannabispirone	Isolated from an extract of a high potency cannabis variety using normal phase chromatography followed by C_18_-HPLC	(Radwan et al., 2008)
		Isocannabispiradienone	Obtained from the dichloromethane extract of decarboxylated *C. sativa* hemp that was subjected to C_18_ flash chromatography, followed by silica gel gravity column chromatography and HPLC.	(Ross S and ElSohly M, 1995)
		*α*-cannabispiranol		
		Cannabispirketal	obtained from the leaves of *C. sativa*. isolated from an ethanolic extract	(T. T. Guo et al., 2017)
		glycoside, *α*-cannabispiranol−4′-O-β-glucopyranose		
		prenylspirodienone	isolated by extensive NMR and ESI-MS analysis.	(Nalli et al., 2018)
	Dihydrostilbenes (12 compounds)	3-[2-(4-hydroxyphenyl)-ethyl]−5-methoxyphenol	Isolated and identified from *C. sativa*	(Turner et al., 1980)
		3-[2-(3-hydroxy−4-methoxyphenyl)-ethyl]−5-methoxyphenol		
		3-[2-(3-isoprenyl−4-hydroxy−5-methoxy-phenyl)-ethyl]−5-methoxyphenol		
		canniprene		
		cannabistilbene I	Isolated from the polar acidic fraction of a Panamanian variant of *C. sativa* grown at the University of Mississippi.	(H. N. ElSohly et al., 1984)
		cannabistibene II		
		3,4′,5-trihydroxy-dihydrostilbene	Isolated from the ethanol extract of a hashish sample	(El-Feraly, 1984)
		α,α′-dihydro−3′,4,5′-trihydroxy−4′-methoxy−3-isopentenylstilbene	Isolated from the leaves of *C. sativa* grown in Yunnan Province, China. applied multiple chromatographic techniques in the isolation and purification of compounds 149–153, such as column chromatography over silica gel cc, ODS C_18_ Si gel column chromatography, Sephadex column chromatography, and preparative HPLC.	(Guo et al., 2018)
		α,α′-dihydro−3,4′,5-trihydroxy−4-methoxy−2,6-diisopentenylstilbene		
		,α′-dihydro−3′,4,5′-trihydroxy−4′-methoxy−2′,3-diisopentenylstilbene		
		α,α′-dihydro−3,4′,5-trihydroxy−4,5′-diisopentenylstilbene		
		combretastatin B−2		
	Dihydrophenanthrenes (7 compounds)	cannabidihydrophenanthrene (cannithrene 1)	Isolated from Thailand cannabis	(Crombie and Crombie, 1982; Shoyama and Nishioka, 1978)
		cannithrene 2		
		4,5-dihydroxy−2,3,7-trimethoxy−9,10-dihydrophenanthrene	Isolated from an ethanolic extract of a high potency cannabis variety grown in Mississippi using a combination of normal and reversed phase chromatographic techniques	(Radwan et al., 2008)
		4-hydroxy−2,3,6,7-tetramethoxy−9,10-dihydrophenanthrene		
		4,7-dimethoxy−1,2,5-trihydroxyphenanthrene		
		1,4-phenanthrenequinone, denbinobin	from an acetone extract of *C. sativa* chemotype (CARMA) after fractionation and column chromatography. Denbinobin (159) was purified by crystallization from ether	(Sánchez-Duffhues et al., 2008)
		2,3,5,6-tetramethoxy 9,10-dihydrophenanthrenedione	Isolated from the leaves and branches of *C. sativa*	(Cheng et al., 2010)
	Simple phenols (7 compounds)	eugenol	detected in the essential oil of Cannabis	(Malingre et al., 1975, Turner et al., 1980)
		methyleugenol		
		iso-eugenol		
		trans-anethol		
		*cis*-anethol		
		Vanillin	Isolated and identified from hemp pectin using silica gel column chromatography and Identified via^1^H NMR,^13^C NMR, and ESI-MS spectroscopic methods	(Chen et al., 2012)
		Phloroglucinol β-D-glucoside	Identified from the stem exudate of greenhouse-grown *C. sativa* by TLC, but its aglycone (phloroglucinol) was isolated after acid hydrolysis of the exudate.	(Hammond and Mahlberg, 1994)
Flavonoids (34 compounds)	Orientin	Orientin-O-glucoside	Isolated from *C. sativa* were reviewed by turner et al. in 1980	(Turner et al., 1980)
		Orientin−7-O glucoside		
		Orientin −7-Oglucoside		
		Orientin −7-O-rhamnoglucoside		
	Vitexin	Vitexin	Identified from Canadian cannabis plants grown from seeds, where the authors used TLC, a hydrolytic test and UV spectroscopic analysis to determine their chemical structures	(Clark and bohm, 1979)
		Vitexin-O-glucoside	Isolated from *C. sativa* were reviewed by Turner et al., 1980	(Turner et al., 1980)
		Vitexin−7-O-glucoside		
		Vitexin−7-O-rhamnoglucoside		
		Cytisoside	Identified from Canadian cannabis plants grown from seeds, where the authors used TLC, a hydrolytic test and UV spectroscopic analysis to determine their chemical structures	(Clark And Bohm, 1979)
		Cytisoside-glucoside		
	Isovitexin	Isovitexin	Isolated from *C. sativa* were reviewed by Turner et al., 1980	(Turner et al., 1980)
		Isovitexin-O-glucoside		
		Isovitexin−7-O-glucoarbinoside		
		Isovitexin−7-O-rhmnoglucoside		
	Apigenin	Apigenin−7-O-glucoside		
		Apigenin−7-O-glucoside		
		Apigenin−7-O-p-coumaroylglucoside		
		6-prenylapigenin		
		Apigenin−6,8-di-glucopyranoside	Isolated from the methanolic extract of hemp	(Cheng et al., 2008)
	Luteolin	Luteolin -C-glucuronid	Isolated from *C. sativa* were reviewed by Turner et al., 1980	(Turner et al., 1980)
		Luteolin−7-O-glucuronid		
		Canniflavin A	Isolated from the ethanolic extract of *C. sativa*. The structures were elucidated by using UV,^1^H NMR and^13^C NMR spectroscopic techniques	(Barrett et al., 1986, Crombie and Crombie, 1982)
		Canniflavin B		
		Canniflavin C	Isolated from a high potency variety of *C. sativa* grown in Mississippi polar fractions by using combination of various chromatographic techniques, such as VLC, silica gel column chromatography, and RP-HPLC	(Radwan et al., 2008)
		Chrysoeriol		
	Kaempferol	Kaempferol−3-O-digluside	Isolated from *C. sativa* were reviewed by turner et al. in 1980	(Turner et al., 1980)
		Quercetin−3-O-glucoside		
		Quercetin−3-O-diglucoside		
		kaempferol−3-O-sophoroside	Isolated from the pollen grains of the male plants of a Mexican variety of *C. sativa* that was cultivated at the University of Mississippi.	(Ross et al., 2005)
		quercetin−3-O-sophoroside		
		Rutin	Isolated for the first time from hemp pectin. The ethanolic extract was purified by macroreticular resin, silica gel column chromatography, and Sephadex-LH−20. Spectroscopic methods (ESI-MS,^1^H NMR,^13^C NMR) were used for identification of its chemical structure	(Chen, 2012)
	Quercetin.	Quercetin	Identified and quantified in the hydroalcoholic extract of hemp inflorescence from monoecious cultivars grown in Central Italy. Four cultivars (Ferimon, Uso−31, Felina 32 and Fedora) were analyzed at four stages of growth from flowering to ripening using HPLC-PDA.	(di Giacomo et al., 2021; Ingallina et al., 2020)
		Naringenin		
		Naringin		
Terpenes (120 compounds)	61 monoterpenes (c_10_ skeleton)	acyclic monoterpene myrcene	Identified in the essential oil of fresh, wild *C. sativa* from Canada	(El-Feraly et al., 1977, Simonsen and Todd, 1942)
		*p*-cymene	Obtained from the low boiling point terpene fraction of Egyptian hashish	(Simonsen and Todd, 1942)
		1-methyl−4-isopropenyl-benzene or dehydro-*p*-cymene	Obtained from the low boiling point terpene fraction of Egyptian hashish	
		monocyclic monoterpene limonene	Identified in the essential oil of fresh, wild *C. sativa* from Canada	(El-Feraly et al., 1977)
		α-terpinene	Detected from the hydrodistillation of freshly harvested *C. sativa* L. from India The essential oil obtained from the hydrodistillation underwent fractional distillation, yielding five fractions. Fraction 5 was further chromatographed over alumina using petroleum ether, benzene, ether, and alcohol successively as eluents. The fractions collected with petroleum ether were combined and named Fraction 5-A, while the fractions collected with benzene as the solvent system were combined and collectively known as Fraction 5-B.	(Nigam et al., 1965)
		β-phellandrene		
		γ-terpinene		
		α-terpinolene		
		α-pinene		
		β-pinene		
		camphene		
		linalool		
		α-terpineol		
		terpinene−4-ol		
		linalool oxide		
		sabinene hydrate		
		*cis*-β-ocimene	Dutch and Turkish cannabis volatile oil samples were compared by capillary gas chromatography The volatile oils were prepared by two methods: hydrodistillation or through nitrogen extraction	(Bercht C et al., 1971; Lousberg and Salemink, 1973)
		*trans*-β-ocimene		
		α-phellandrene		
		Δ^3^-carene		
		Δ^4^-carene		
		sabinene		
		α-thujene		
		*m*-mentha−1,8-(9)-dien−5-ol	Identified from the volatile oil of *Cannabis*	(Strömberg, 1976)
		namely 2-methyl−2-heptene−6-one	Samples were prepared by weighing 1 g of each, placing it in a microvial, and heating at 65°C for 1 hour. Then, 5 mL of headspace air was withdrawn with a gas-tight syringe and directly injected into the gas chromatograph.	(Hood et al., 1973)
		fenchyl alcohol		
		borneol		
		nerol	The volatile oil of *C. sativa* of Mexican origin was prepared and analyzed using GC-MS (Gas Chromatography-Mass Spectrometry).	(Smith and Kempfert, 1977)
		geraniol		
		carvacrol		
		1,8-cineol		
		4-cineol		
		camphor		
		piperitenone		(Strömberg, 1974)
		3-phenyl−2-methyl-prop−1-ene	Cannabis essential oil was extracted using steam distillation and a lighter-than-water volatile oil apparatus. The oil was then analyzed by GC-MS and GC-FID to identify monoterpenes.	(Malingre et al., 1975)
		23 oxygenated hydrocarbons, namely citral B		
		citronellol		
		geranyl acetone		
		carvone		
		pulegone		
		dihydrocarvone		
		β-terpineol		
		dihydrocarveyl acetate		
		*p*-cymene−8-ol		
		β-cyclocitral		
		safranal		
		*cis*-linalool oxide		
		perillene		
		sabinol		
		thujyl alcohol		
		piperitone oxide		
		piperitenone oxide		
		fenchone		
		bornyl acetate		
		camphene hydrate		
		α-pinene oxide		
		pinocarveol		
		pinocarvone		
		ipsdienol		(Ross and ElSohly, 1996)
		*cis*-carveol		
		*cis*-sabinene hydrate		
	Sesquiterpenes (51 compounds)	α-caryophyllene (α-humulene)	Obtained with analysis of the higher boiling point fraction of Egyptian hashish,	(Simonsen and Todd, 1942)
		α-caryophyllene	Identified in the volatile oil of fresh *C. sativa*, through GC analysis	(Martin Et Al., 1961)
		β-caryophyllene		
		caryophyllene oxide	Identified in the volatile oil of Indian *C. sativa* in 1965	(Nigam et al., 1965)
		curcumene		
		α-*trans*-bergamotene		
		α-selinene		
		β-farnesene		
		longifolene	Reported from the analysis of the volatile oil of *C. sativa*	(Stahl and Kunde, 1973)
		humulene epoxide I		
		humulene epoxide II		
		caryophyllene alcohol (caryophyllenol)		
		β-bisabolene	Reported in one study, obtained with analysis of headspace volatiles, volatile oil, and samples of marijuana from Customs’ seizures	(Hood et al., 1973)
		allo-aromadendrene	Reported for the first time from the essential oil of *C. sativa* grown in Mexico in 1974. The compounds were identified using both GC/FID and GC/MS	(Strömberg, 1974)
		calamenene		
		α-copaene		
		nerolidol	Identified in the volatile oil of *C. sativa* from Mexico through GC-MS analysis by Bercht and Paris in 1974	(Smith and Kempfert, 1977)
		α-gurjunene	Detected for the first time in *C. sativa* resin Using GC/MS and GC retention time	(Strömberg, 1974)
		iso-caryophyllene	Identified in 1975 in the essential oil of *Cannabis*, and later confirmed by the same research group to be present in the essential oil of *C. sativa* in 1978 by GC and GC-MS analyses	(Malingre et al., 1975)
		β-selinene		
		selina−3,7(11)-diene		
		selina−4(14),7(11)-diene		
		α-gurjunene	Reported by the same previous study in 1978 for the first time using GC-MS analyses of the essential oil of *Cannabis*	(Malingre et al., 1975)
		α-bisabolol		
		α-cedrene		
		α-cubebene		
		δ-cadinene		
		epi-β-santalene		
		farnesol		
		γ-cadinene		
		γ-elemene		
		γ-eudesmol		
		guaiol		
		ledol		
		*trans*-*trans*-α-farnesene		
		(*Z*)-β-farnesene		
		farnesyl acetone		
		α-cadinene	In 1996, 14 new sesquiterpenes were identified,	(Ross and ElSohly, 1996)
		α-*cis*-bergamotene		
		α-eudesmol		
		α-guaiene		
		α-longipinene		
		α-ylangene		
		β-elemene		
		β-eudesmol		
		epi-α-bisabolol		
		γ-*cis*-bisabolene		
		γ-curcumene		
		γ-muurolene		
		γ-*trans*-bisabolene		
		germacrene-B	Detected for the first time from hemp essential oil and was quantified by GC-MS	(Ingallina et al., 2020, Menghini et al., 2021)
		clovandiol	Identified in organic extract of cannabis infloresence of Ferimon and Uso−31 cultivars	
	Diterpenes	Diterpenes Phytol	Identified by GC-MS	(Ingallina et al., 2020, Malingre et al., 1975)
		neophytadiene		
	Triterpenes	friedelin (friedelan−3-one)	1971, analysis of the ethanolic extract of *Cannabis* roots via spectral data and comparison with authentic samples	(Slatkin et al., 1971)
		epifriedelanol		
	Miscellaneous terpenes	vomifoliol	Isolated from Dutch hemp Both componds were identified from the stems and leaves of the plant through isolation, spectral data comparison, and synthesis from (+)-α-ionone.	(Smith and Kempfert, 1977)
		dihydrovomifoliol		
		β-ionone	were identified from the volatile oil of *C. sativa*	(Malingre et al., 1975)
		dihydroactinidiolide		
Alkaloids	Spermidine alkaloids	cannabisativine	Identified anhydrocannabisativine (322) in 15 different *Cannabis* variants using TLC eluted with chloroform: acetone: ammonia (1:1:1) [	(Elsohly et al., 1978)
		anhydrocannabisativine	Isolated from the dry leaves and small stems of cannabis of the Mexican variety grown in Mississippi through a series of acid–base extractions and silica-gel chromatography.	

#### 1.1.1 Cannabinoids (11 subcategory) (Fig. 1)

The term "cannabinoid" broadly refers to a group of compounds with a characteristic C21 terpenophenolic backbone, including synthetic cannabinoids, endocannabinoids, and phytocannabinoids that interact with cannabinoid receptors (Pertwee, 2005). Initially, the term was used to describe a set of oxygenated aromatic hydrocarbon metabolites from marijuana, now known as phytocannabinoids. Cannabinoids are molecules that interact with the endocannabinoid system (ECS) in the body. The discovery of phytocannabinoids began in 1964 when Raphael Mechoulam and Yechiel Gaoni isolated THC, identifying it as the primary psychoactive compound in cannabis (Mechoulam and Gaoni, 1965, Pertwee, 2006). This discovery led to the identification of the ECS, named for its interaction with cannabinoids. In 1988, Devane et al. identified the first cannabinoid receptor (CB1) in the brain (Devane et al., 1988), followed by the isolation of the first endocannabinoid, anandamide, in 1992 (Devane et al., 1992). Named after the Sanskrit word for "bliss," anandamide revealed the natural production of cannabinoids in the human brain, distinct from those in cannabis (Crocq, 2020).

**Fig. 1:**
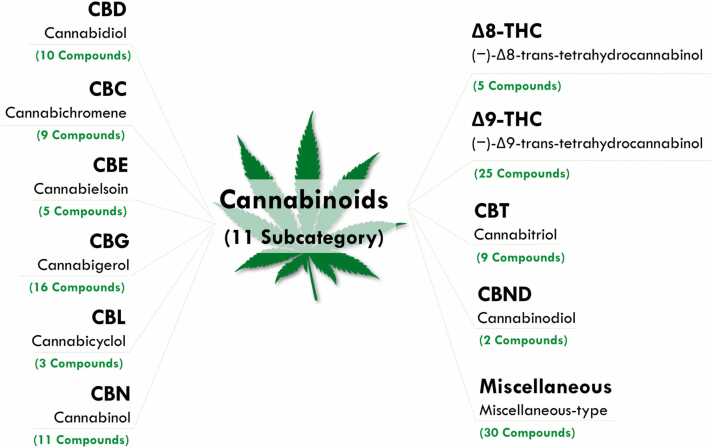
Cannabinoid Sub-Categories Found in Cannabis. This figure illustrates the diversity of cannabinoids in cannabis, classified into 11 sub-categories. These include cannabidiol (CBD), cannabichromene (CBC), cannabielsoin (CBE), cannabigerol (CBG), cannabicyclol (CBL), cannabinol (CBN), cannabinodiol (CBND), cannabitriol (CBT), (−)-Δ8-trans-tetrahydrocannabinol (Δ8-THC), (−)-Δ9-trans-tetrahydrocannabinol (Δ9-THC), and miscellaneous-type cannabinoids (Radwan et al., 2021). Each category is characterized by varying numbers of compounds, such as Δ9-THC with 25 compounds, CBG with 16 compounds, and CBD with 10 compounds. These cannabinoids exhibit a broad spectrum of pharmacological activities, including neuroprotective, anti-inflammatory, and antioxidant properties, which are relevant AD research.

As the significant potential of the ECS continues to emerge, interest in these molecules has grown, leading to extensive research. Cannabis phytocannabinoids, a key group within this category, can be categorized into 11 (Fig. 1) distinct sub-categories: cannabichromene (CBC), CBD, cannabielsoin (CBE), cannabigerol (CBG), cannabicyclol (CBL), cannabinol (CBN), cannabinodiol (CBND), cannabitriol (CBT), (−)-Δ8-trans-tetrahydrocannabinol (Δ8-THC), Δ9-THC, and miscellaneous-type cannabinoids (Radwan et al., 2021) (Fig. 1). Among these 11 sub-categories, the most extensively studied are the psychotropic cannabinoids, with THC (Δ9-THC) being the most notable, followed by CBN and Δ8-THC. Non-psychotropic cannabinoids such as CBD, CBC, and CBG are also of significant interest due to their therapeutic potential. These six compounds: THC, CBD, CBN, CBC, CBG, and Δ8-THC, are often referred to as "the major cannabinoids" or "the big four" due to their prevalence and importance in cannabis research (Atakan, 2012, Lewis et al., 2017).

The Table 1 will detail these cannabinoids: with sub-categories, including the compounds types within each category and the methods used for their extraction.

#### 1.1.2 Non cannabinoids (Fig. 2)

**Fig. 2:**
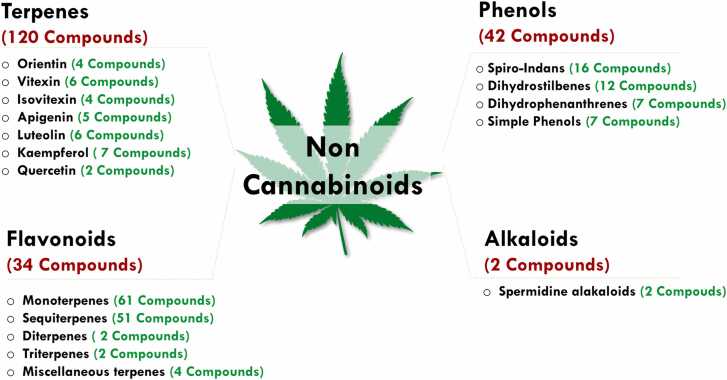
Non-Cannabinoid Sub-Categories Found in Cannabis. This figure highlights the diversity of non-cannabinoid compounds in cannabis, categorized into terpenes (120 compounds), flavonoids (34 compounds), phenols (42 compounds), and alkaloids (2 compounds). Each category is further divided into subtypes, such as orientin, vitexin, and quercetin (terpenes), or spiro-indans and dihydrostilbenes (phenols) (Adapted from Radwan et al., 2021). These bioactive compounds contribute to cannabis' therapeutic potential by modulating inflammation, oxidative stress, and neuroprotection, complementing cannabinoids in AD research.

In addition to cannabinoids, over 400 non-cannabinoid constituents have been isolated and identified from the cannabis plant. These non-cannabinoid compounds belong to various chemical sub-categories (Fig. 2), including phenols, flavonoids, terpenes, and alkaloids (ElSohly and Slade, 2005; Turner et al., 1980). The non-cannabinoid components of cannabis were first identified during the early chemical analyses of the plant in the mid-20th century, but they were initially overshadowed by the more psychoactive and widely studied cannabinoids (Radwan et al., 2021). Terpenes, which contribute to cannabis's distinctive aroma, were among the first non-cannabinoid compounds to be characterized. Despite this, these compounds were largely considered secondary to the cannabinoids and were not deeply investigated for their therapeutic potential (Sommano et al., 2020).

Non-cannabinoid molecules belong to a broad spectrum of secondary metabolites extracted from plants. Several criteria are used to classify these molecules, including chemical structure (such as the presence of rings or sugars), composition (whether they contain nitrogen), solubility in organic solvents or water, and biosynthetic pathways (Lowe et al., 2021). Among these, the biosynthetic pathway is the most commonly used criterion for grouping secondary metabolites in plants. Based on this approach, non-cannabinoid compounds are divided into four major groups: terpenes, phenolic compounds, flavonoids, and alkaloids (Satish et al., 2020). For example, phenolic compounds are natural metabolites primarily derived from the shikimate/phenylpropanoid pathway, which produces phenylpropanoids. These compounds are characterized by an aromatic ring with one or more hydroxyl groups (Santos-Sánchez et al., 2019).

With further subdivisions based on chemical structure and functional groups. Phenols are divided into subgroups such as spiro-indans, which feature a distinctive spiro-linked ring system, and dihydrostilbenes, characterized by two phenyl rings connected by an ethylene bridge, as detailed by (Bercht et al., 1973; Turner et al., 1973). Flavonoids include cannabis-specific cannflavins, known for their anti-inflammatory properties, and more common plant flavonoids like quercetin, recognized for antioxidant activity, as outlined by (Guo et al., 2018). Terpenes are categorized into monoterpenes, smaller molecules like myrcene, and sesquiterpenes, larger molecules such as β-caryophyllene, both distinguished by the number of isoprene units they contain, as reported by (ElSohly and Slade, 2005). Alkaloids are divided into simple and complex types based on the presence of nitrogen atoms and the complexity of their ring structures, as described in studies like those of (Radwan et al., 2008).

Interest in non-cannabinoid compounds began to increase in the late 20th and early 21st centuries, driven by the discovery of the "entourage effect." This concept suggests that the therapeutic effects of cannabis are not solely due to individual cannabinoids but also to the synergistic interactions between cannabinoids and non-cannabinoid compounds. Researchers began to explore how terpenes and flavonoids might modulate the effects of cannabinoids, enhance bioavailability, and exert their own therapeutic properties (Ferber et al., 2020).

Advances in extraction technologies, such as supercritical CO_2_ extraction, have allowed for the more precise isolation of non-cannabinoid compounds from cannabis. These methods have enabled researchers to study these compounds more rigorously, leading to discoveries about their anti-inflammatory, neuroprotective, and antioxidant properties. For example, terpenes like β-caryophyllene have been found to activate cannabinoid receptors independently of THC, offering potential anti-inflammatory and neuroprotective effects (Cheng et al., 2014a). Flavonoids, including cannflavins, have demonstrated strong anti-inflammatory actions, surpassing even aspirin in some models (Abdel-Kader et al., 2023)

Table 2 provides an overview of these chemical sub-categories in cannabis, including their compounds types and the methods used for their extraction.

### 1.2 Studies exploring the effects of cannabis extracts on AD

Cannabis (*Cannabis sativa* L.) is renowned for its rich chemical diversity across various biogenetic categories. While the plant contains unique phytochemicals, many of these compounds belong to chemical categories shared with other plants. The therapeutic potential of cannabis, particularly in the context of neurodegenerative diseases like Alzheimer's, has garnered significant attention (Abate et al., 2021).

Two main research approaches have emerged in the study of cannabis compounds for AD: one focusing on isolated, highly purified molecules, and the other utilizing complex extracts with multiple known or unknown compounds. The latter approach often emphasizes the entourage effect mentioned earlier (Ferber et al., 2020). For instance, the combination of THC and CBD has demonstrated enhanced therapeutic outcomes compared to either compound alone (Christensen et al., 2023).

Much of the therapeutic interest in cannabis centers around its modulation of the ECS through phytocannabinoids, as it has become increasingly clear that the ECS is a crucial regulator of various biological responses (Lu and Mackie, 2016). Phytocannabinoids can increase the levels of endocannabinoids such as anandamide (AEA) and 2-arachidonoylglycerol (2-AG), which bind to endocannabinoid receptors (CB1Rs) in the nervous system, particularly at GABAergic nerve terminals. This interaction enhances dopamine concentration and transmission, contributing to antipsychotic and antidepressive effects observed in animal models (Bloomfield et al., 2016, Bloomfield et al., 2019). These effects are likely mediated by interactions with TRPV1 and serotoninergic receptors (5-HT1A), which play essential roles in emotional regulation, stress response, and neuroprotection (Sales et al., 2018).

Moreover, cannabinoids have been shown to activate Peroxisome Proliferator-Activated Receptor Gamma (PPARγ), leading to microglial activation and reduced expression of inflammatory genes, further exerting neuroprotective effects (Nadal et al., 2017). This broad interaction with multiple therapeutic targets underscores the potential of cannabinoids in modulating the pathogenesis of neurodegenerative diseases (Dos Reis Rosa Franco et al., 2021).

With the growing understanding of the ECS, a wide array of molecules has emerged, capable of modulating ECS activity and showing potential therapeutic benefits in AD (Karl et al., 2012). These compounds include endocannabinoid reuptake inhibitors, which extend the action of naturally occurring endocannabinoids, and enzyme inhibitors that prevent their breakdown, thereby enhancing their effects. Furthermore, synthetic molecules that selectively target ECS receptors, either by activating or blocking them, have been developed to fine-tune the system's activity. These ECS modulators are particularly promising in Alzheimer's research, as they can impact key processes such as neuroinflammation, synaptic plasticity, and neuronal survival, which are crucial in the disease's progression. Although these compounds are still under investigation, they hold significant potential for alleviating symptoms or slowing the advancement of Alzheimer's by harnessing the body's endogenous cannabinoid system (Karl et al., 2012).

In addition to cannabinoids, non-cannabinoid phytochemicals have also demonstrated therapeutic potential. Notable among these are flavonoids such as cannflavin A-C, the stilbenoid canniprene, and a range of terpenes (Andre et al., 2016). Cannflavins, which can constitute up to 1 % of cannabis leaf material, possess strong anti-inflammatory profiles, while canniprene targets 5-lipoxygenase, a key enzyme involved in neuroprotection (Izzo et al., 2020; Yelanchezian et al., 2022). Although these minor compounds are less studied compared to conventional flavonoids, recent evidence suggests that they also exhibit neuroprotective effects, such as inhibiting Aβ aggregation (Hole and Williams, 2021).

Terpenes, another significant sub-category of cannabis phytochemicals. Common terpenes like limonene, α-pinene, and β-caryophyllene have demonstrated neuroprotective properties, including the ability to stimulate antioxidant defenses, limit ROS-induced apoptosis, and inhibit Aβ aggregation (Porres-Martínez et al., 2016). Additionally, some terpenes like β-caryophyllene and α-bisabolol have shown promise in reducing neurodegenerative effects via cannabinoid receptor-independent pathways (Porres-Martínez et al., 2016).

Given the significant scientific interest in cannabis, numerous studies have explored the effects of its compounds on AD. Recognizing the abundance of references in this area, we deemed it essential to classify these studies using a suitable categorization system. In the first part of our research, we categorized cannabis-related compounds into cannabinoids and non-cannabinoids.

#### 1.2.1 Cannabinoids effect on AD

We first focused on cannabinoid compounds, distinguishing between natural and synthetic ones or ECS modulators.

**Natural compounds:** include all cannabinoids that affect the ECS, whether they are derived from cannabis plants or produced endogenously within the body (endocanabinoids). Importantly, we considered the chemical structure of these compounds to be natural, regardless of whether they were isolated from natural sources or chemically synthesized.

On the other hand, **synthetic compounds or ECS modulators:** are chemically designed to mimic, enhance, or inhibit the effects of natural cannabinoids. These compounds may replicate the effects of their natural counterparts, amplify them, or act as inhibitors.

In this section, we will present this sub-category of compounds and their effects within the context of AD (Table 3).

**Table 3:** Findings highlight the potential neuroprotective, anti-inflammatory, antioxidant, and cognitive-enhancing properties of natural and synthetic cannabinoids in AD, emphasizing their promise in mitigating neuroinflammation, promoting synaptic plasticity, and reducing oxidative stress.

**Cannabinoids: Natural compounds**			
Types of Compounds	Experimental Substrate and Method of Administration	Study Results	References
phytocannabinoids Δ9-Tetrahydrocannabinol (THC)	- **In vivo** Male C57BL/6 J mice - Implanted osmotic minipumps releasing THC (3 mg per kg bodyweight per day)	**Neuroprotection**: - Low-dose Δ9-THC reversed age-related cognitive decline in 12- and 18-month-old mice. - THC-treated 12-month-old mice exhibited cognitive performance similar to 2-month-old untreated mice. **Synaptic Plasticity**: ⬆️ Treatment increased expression of synaptic markers and hippocampal spine density.	(Bilkei-Gorzo et al., 2017
phytocannabinoids Δ9-Tetrahydrocannabinol (THC)	**- In vivo** - Tg (Thy1-EGFP) MJrs/J (GFP-M) male mice (3-month-old and 18-month-old) - Chronic low-dose THC administration via osmotic pump (3 mg/kg/day for 28 days)	**Neuroprotection**: ⬆️ In old mice, THC improved dendritic spine stability, leading to a long-lasting increase in spine density, potentially enhancing cognitive function. **Synaptic Plasticity**: ⬇️ In young mice, THC transiently increased spine turnover and destabilized the spines, which could impair cognitive function.	(Komorowska-Müller et al., 2023)
phytocannabinoids CBD: Cannabidiol	- **In vitro** -Cultured rat pheochromocytoma PC12 cells -Incubation with cannabidiol (10(^−7^)−10(−4)m) prior to beta-amyloid peptide exposure	**Neuroprotection**: CBD improved cell survival in β-amyloid-exposed PC12 cells. **Antioxidant**: ⬇️ CBD reduced ROS, lipid peroxidation, caspase 3, DNA fragmentation, and intracellular calcium. **Anti-Apoptotic**: CBD has neuroprotective, antioxidant, and anti-apoptotic effects.	(Iuvone et al., 2004)
phytocannabinoids CBD: Cannabidiol	**In vitro** - PC12 neuronal cells -Incubation with Cannabidiol 10^−7^ and 10^−5^ M.	**Neuroprotection**: Cannabidiol rescues PC12 neuronal cells from Aβ-induced toxicity. **Proteopathy (Tau Hyperphosphorylation)**: ⬇️ Cannabidiol inhibits tau protein hyperphosphorylation. **Wnt/β-Catenin Pathway Modulation**: The neuroprotective effect is mediated through the rescue of the Wnt/β-catenin pathway.	(Esposito, De Filippis, Carnuccio, et al., 2006)
phytocannabinoids CBD: Cannabidiol	**In vitro** - PC12 neuronal cells -Incubation with CBD (10^−6^) and 10(^−4^) M	**Neuroinflammation**: ⬇️ Cannabidiol inhibits nitrite production and iNOS protein expression induced by Aβ in a concentration-dependent manner. **Wnt/β-Catenin Pathway Modulation**: The neuroprotective effect is mediated through the inhibition of p38 MAP kinase phosphorylation and NF-κB activation. **Anti-Inflammatory**: Cannabidiol's anti-inflammatory properties suggest its potential in preventing Aβ-induced neurodegeneration with low toxicity in humans.	(Esposito, De Filippis, Maiuri, et al., 2006)
phytocannabinoids CBD: Cannabidiol	**In vitro** -SHSY5Y^APP+^ neurons stably transfected with APP695. -Incubation with CBD (10^−9^ and 10^−6^ M)	**Proteopathy (Amyloid Beta Production)**: ⬇️ Cannabidiol (CBD) induced the ubiquitination of amyloid precursor protein (APP), leading to a significant reduction in APP full-length protein levels and a subsequent decrease in Aβ production in SHSY5YAPP+ neurons. **Anti-Apoptotic**: ⬆️ CBD promoted increased neuronal survival by reducing long-term apoptotic rates in SHSY5YAPP+ cells. **PPARγ Modulation**: All observed effects of CBD were dependent on the selective activation of peroxisome proliferator-activated receptor-γ (PPARγ).	(Scuderi et al., 2014)
phytocannabinoids CBD: Cannabidiol	**In vivo:** -Adult male Sprague-Dawley rats (300–350 g). - rats were i.p. administered for 15 days with: CBD 10 mg/kg **In vitro:** -Rat primary astroglia cultures obtained from newborn Sprague-Dawley rats treated with 1 µg/mL Aβ (1–42) in the presence or absence of CBD (10^−9^–10^−7^ M)	⬇️ CBD's neuroprotective effects in rat AD models are mediated through PPARγ, as blockade of this receptor significantly reduces CBD's impact on reactive gliosis and neuronal damage. **Neurogenesis**: ⬆️ CBD stimulates hippocampal neurogenesis via its interaction with PPARγ, highlighting the receptor's crucial role in mediating CBD's actions.	(Esposito et al., 2011)
phytocannabinoids CBD	**In vivo:** - 3–5-months old C57BL/6 J mice (35–40 g) injected with 10 ng of A*β* (1–42) in dorsal hippocampus - CBD treatment: daily intraperitoneal injection with (2.5 or 10 mg kg^−1^) for 7 days	**Neuroinflammation**: ⬇️ CBD dose-dependently inhibited GFAP mRNA and protein expression in Aβ-injected animals. ⬇️ CBD reduced iNOS and IL−1β protein expression and their associated NO and IL−1β release. **Anti-Inflammatory**: Results confirm CBD's in vivo anti-inflammatory actions.	(Esposito et al., 2007)
phytocannabinoids CBD	**In vivo:** - male AβPPSwe/PS1ΔE9 (AβPP × PS1) - AD transgenic mice were treated orally from 2.5 months of age with CBD (20 mg/kg) daily for 8 months.	**Behavioral Comorbidity of AD**: ⬆️ CBD treatment prevented the development of social recognition deficits in AD transgenic mice. **Anxiety and Learning**: No impact on anxiety levels or associative learning abilities in the mice. **Amyloid Load and Oxidative Damage**: No changes in amyloid load or oxidative damage were observed with CBD treatment. **Neuroinflammation**: Subtle impact on neuroinflammation, cholesterol levels, and dietary phytosterol retention; effects require further investigation.	(Cheng et al., 2014c)
phytocannabinoids CBD	**In vivo:** - Transgenic mouse model of AD (AβPPxPS1 mice) - Intraperitoneal injections (50 mg/kg CBD daily for 3 weeks)	**Behavioral Comorbidity of AD**: ⬆️ CBD treatment restored social recognition memory and spatial learning deficits in the mice. **Amyloid Load**: ⬇️ CBD tended to reduce insoluble Aβ40 levels in the hippocampus. **Neuroinflammation**: No effect on neuroinflammation, neurodegeneration, or PPARγ markers in the cortex.	(Watt et al., 2020)
phytocannabinoids CBD	**In vivo:** - Transgenic AD mouse model (APPswe/PS1∆E9 mice) - Intraperitoneal injections (20 mg/kg CBD daily for 3 weeks)	**Behavioral Comorbidity of AD**: ⬆️ CBD treatment reversed impairments in social recognition and novel object recognition in AD transgenic mice. **Anxiety**: No effect on anxiety-related behaviors in the treated mice.	(Cheng et al., 2014b)
Endocannabinoids: 2-rachidonoylglycerol (2-AG)	**In vitro:** -Hippocampal neurons in culture -Direct application of 2-AG to cultured hippocampal neurons.	**Neuroprotection**: ⬆️ Exogenous 2-AG significantly protected hippocampal neurons against β-amyloid (Aβ)-induced neurodegeneration and apoptosis. ⬆️ MAGL inhibitors URB602 and JZL184, which elevate endogenous 2-AG levels, also significantly reduced Aβ-induced neurodegeneration and apoptosis. **Cannabinoid Receptor Modulation**: ⬇️ The neuroprotective effect of 2-AG was blocked by SR141716 (a selective CB1R antagonist) but not by SR144528 (a selective CB2R antagonist) or capsazepine (a selective TRPV1 receptor antagonist). **Signaling Pathways**: The neuroprotective effects of 2-AG are mediated via CB1R-dependent suppression of ERK1/2 and NF-κB phosphorylation and COX−2. **Therapeutic Potential**: Elevation of endogenous 2-AG by inhibiting its hydrolysis has potential as a novel therapeutic approach for preventing, ameliorating, or treating AD.	(Chen et al., 2011)
phytocannabinoids (Δ9-THC and CBD)	**In vivo** -wild-type (WT) and transgenic (APP/PS1) mice aged 3 and 12 months - ip injection of 0.75 mg/kg for each cannabinoid once daily for 5 weeks - Δ9-THC-enriched botanical extract (67 % Δ9-THC, 0.8 % CBD) - CBD-enriched extract (62.7 % CBD, 3.6 % Δ9-THC)	**Behavioral Comorbidity of AD**: ⬆️ Δ9-THC and CBD botanical extracts reduce memory impairment in advanced-stage AβPP/PS1 mice. **Amyloid Processing and Glial Reactivity**: No alteration in Aβ processing or glial reactivity. **Cognitive Function in Healthy Mice**: No impact on cognitive impairment in healthy aging wild-type mice. **Neurochemical Changes**: ⬆️ Positive effects in aged AβPP/PS1 mice are linked to reduced GluR2/3 and increased GABA-A Rα1 levels in treated animals.	(Aso et al., 2016, aso et al., 2016)
phytocannabinoids (Δ9-THC and CBD)	**In vivo** - 19–20-month-old mice - Inhalation of vaporized cannabis with 38-L exposure chamber (60 cm × 45 cm × 20 cm), that included a vapor inflow tube and several small air outflow holes	**Pain Relief**: ⬆️ Chronic Δ9-THC use provided effective pain relief. **Anxiolytic and Cognitive Effects**: ⬇️ Chronic Δ9-THC use led to diminished anxiolytic and cognitive effects over time, affecting midbrain dopaminergic volume and gray matter. **Behavioral Impact and Network Connectivity**: No effect on behavior from CBD. ⬆️ CBD improved network connectivity, with lasting changes observed after drug cessation.	(Sadaka et al., 2023)
phytocannabinoids (Δ9-THC and CBD)	**In vivo** - Male adult C57BL/6JArc mice - 21 daily intraperitoneal injections -Δ9-THC (0.3, 1, 3, or 10 mg/kg) -CBD (1, 5, 10, or 50 mg/kg)	**Anxiety**: ⬆️ Δ9-THC induced increased anxiety. ⬆️ Chronic CBD exhibited anxiolytic effects, improving anxiety. **Motor Activity**: ⬇️ Δ9-THC reduced motor activity. ⬇️ Chronic CBD reduced hyperlocomotion. **Prepulse Inhibition**: ⬆️ Δ9-THC enhanced prepulse inhibition. **Psychoactive Side Effects**: Chronic CBD improved anxiety and reduced hyperlocomotion without Δ9-THC’s psychoactive side effects.	(Long et al., 2010)
**phytocannabinoids**: (CBG) and (CBD)	**In vitro** - NSC−34 motoneuron-like cell line, differentiated by serum deprivation and treated with all-trans retinoic acid (RA). - applied directly to NSC−34 cells in culture	**Anti-Inflammatory**: ⬇️ CBD (5 µM): Decreased TNF-α levels; increased IL−10 and IL−37 expression. ⬇️ CBG and CBD (5 µM): Reduced NF-kB nuclear factor activation; decreased iNOS expression. **Anti-Oxidant**: ⬆️ CBG and CBD (5 µM): Increased Nrf2 levels. **Anti-Apoptotic**: ⬇️ CBG and CBD (5 µM): Downregulated Bax protein expression; ⬆️upregulated Bcl−2 expression. **PPARγ Modulation**: Effects were mediated via PPARγ.	(Mammana et al., 2019)
**phytocannabinoids**: (CBDA)and (THCA)	**In Vivo**: Aβ1–42-treated mouse model. - Intrahippocampal stereotaxic injection: Aβ1–42, CBDA (6 μM), or THCA (12 μM) administered (3 μL/15 min/mouse) into the hippocampus **In Vitro**: Primary neurons. Cell were Cultures at 6 days were treated with Aβ_1–42_ and/or CBDA or THCA for 24 h,	**Cognitive Function**: ⬆️ CBDA and THCA treatment in Aβ1–42-treated mice improved cognitive function compared to untreated Aβ1–42 mice. **Amyloid-β and Phospho-Tau Levels**: ⬇️ CBDA and THCA treatment decreased hippocampal Aβ and p-tau levels in Aβ1–42-treated mice. ⬇️ CBDA and THCA lowered Aβ and p-tau levels in primary neurons. **Calcium Dyshomeostasis**: ⬇️ CBDA and THCA alleviated calcium dyshomeostasis. **Neuroprotection**: ⬆️ CBDA and THCA exhibited neuroprotective effects.	(Kim et al., 2023)
Phytocannabinoids: (Cannabichromene (CBC) Cannabigerol (CBG) Cannabinol (CBN) Cannabidivarin (CBDV) Cannabidiol (CBD) Δ9-Tetrahydrocannabinol (Δ9-THC)	**In vitro** - PC12 cells. - applied directly to PC12 cells in culture.	**Neuroprotection and Cytotoxicity**: ⬇️ CBD: Inhibited lipid peroxidation but had no significant effect on Aβ toxicity. ⬆️ CBN, CBDV, CBG: Provided neuroprotection against Aβ-induced cytotoxicity. ⬆️ CBC, CBG, CBN, Δ9-THC, CBD, CBDV: Inhibited Aβ1–42-induced neurotoxicity in PC12 cells. **Aβ Aggregation**: ⬇️CBC, CBN, CBDV: Inhibited Aβ aggregation. ⬇️Δ9-THC: Reduced Aβ aggregate density. **Cell Morphology**: ⬇️CBC, CBG: Inhibited morphological changes induced by Aβ1–42. -No alteration by Δ9-THC, CBD, CBDV in Aβ1–42 effects on cell morphology.	(Marsh et al., 2024)
**Cannabinoids: Synthetic Cannabinoids and ECS modulators:** lab-synthesized cannabinoids, distinct from natural phytocannabinoids, designed to mimic or interact with the endocannabinoid system (Simple and combined synthetic cannabinoids).			
Category and Class of Cannabis	Experimental Substrate and - Method of Administration	Study Results	References
synthetic cannabinoid (dronabinol)	**Clinical study** investigate effects of dronabinol in 15 patients15 AD patients	**Behavioral Comorbidity of AD**: ⬆️ Dronabinol treatment decreased the severity of disturbed behavior in patients, with the effect persisting during the placebo period for those who received dronabinol first.	(L Volicer et al., 1997)
synthetic cannabinoid (Nabilone)	**Clinical study** - Human patients with moderate-to-severe AD - Oral administration of Nabilone (target dose: 1–2 mg) over a 14-week period, with a 6-week treatment phase for both Nabilone and placebo, and a 1-week washout between phases	**Behavioral Comorbidity of AD:** ⬆️ Nabilone significantly reduced agitation compared to placebo (CMAI score improved by −4.0 points). **Therapeutic Potential:** ⬆️ Nabilone improved the NPI-NH total score, NPI-NH caregiver distress score, and sMMSE score. ⬆️CGIC improvement was higher during the Nabilone phase (47 %) compared to placebo (23 %), but the difference was not statistically significant. **Cognitive Function:** ⬇️ In the subset of patients who completed the Severe Impairment Battery (SIB), placebo showed better results.	(Herrmann et al., 2019)
synthetic cannabinoid (Nabilone)	**Clinical study** -Human patients with AD - Oral (nabilone 1–2 mg)	**Oxidative Stress and Inflammation:** - The trial found that oxidative stress (4-HNE) and proinflammatory cytokine TNF-α were associated with agitation severity in Alzheimer's patients. **Anti-Inflammatory and Behavioral Comorbidity of AD:** ⬆️Nabilone showed potential anti-inflammatory effects and was associated with reduced agitation severity during its phase of administration.	(M. Ruthirakuhan et al., 2020)
synthetic cannabinoid (Nabilone)	**Clinical study** -Human patients with moderate-to-severe AD -Oral (nabilone, 0.5–2 mg, vs. placebo)	**Behavioral Comorbidity of AD:** ⬆️ Reduction in agitation severity, measured by the Cohen-Mansfield Agitation Inventory. **Therapeutic Potential:** -A safe and efficacious treatment for agitation in AD could increase quality of life, reduce caregiver burden, and avoid the negative impact of untreated agitation on healthcare costs.	(M. T. Ruthirakuhan et al., 2019)
Cannabinoid (CB2) receptor agonist: MDA7	**In vivo** -Rats (injected with amyloid-β (Aβ)(1−40) fibrils into the hippocampal CA1 area) -MDA7 (selective CB2 agonist) Administered intraperitoneally at 15 mg/kg daily for 14 days	**Neuroinflammation**: ⬆️ MDA7 treatment improved CD11b (microglia marker) and GFAP (astrocyte marker) expression. ⬇️ Reduced interleukin−1β secretion and decreased CB2 receptor levels. **Aβ Clearance**: ⬆️ Promoted Aβ clearance. **Cognitive Function**: ⬆️ Restored synaptic plasticity, cognition, and memory. **Therapeutic Potential**: MDA7 is proposed as a promising therapeutic approach for AD.	(J. Wu et al., 2013)
Synthetic cannabinoid receptor (CB1) receptor agonist: (ACEA)	**In vitro** - Double AβPP (swe)/PS1(1dE9) transgenic mice and primary cultures of cortical neurons	**Cognitive Function**: ⬆️ ACEA improved cognitive function in early-stage transgenic mice. **Aβ Aggregation and Toxicity**: ⬇️ ACEA did not alter Aβ levels or aggregation. ⬆️ ACEA reduced Aβ42 toxicity and reversed GSK3β dephosphorylation. **Tau Phosphorylation and Neuroinflammation**: ⬇️ Lower phospho-tau and reduced astroglial response and interferon-γ expression. **Cannabinoid Receptor Modulation**: ACEA shows potential for treating AD by targeting CB1 receptors.	(Aso et al., 2012)
Synthetic cannabinoid receptor (CB1) receptor agonist: (ACEA)	**In vivo** -Rat models (specifically CA1 pyramidal neurons) -Administration via co-treatment with Aβ	**Memory and Cognitive Function:** ⬇️ Aβ peptide (1−42) injections into the prefrontal cortex impaired memory retention and recall in passive avoidance tasks. **Neurodegeneration:** ⬆️ Active caspase−3 levels increased in the hippocampus following Aβ treatment**.** **Neuronal Activity:** ⬇️ Reduced action potential frequency and increased irregularity in CA1 pyramidal neurons after Aβ treatment. ⬇️ Aβ treatment altered both spontaneous and evoked neuronal responses. **Neuroprotection:** ⬆️ Co-treatment with ACEA (CB1 receptor agonist) preserved normal electrophysiological properties of pyramidal cells, demonstrating neuroprotective effects against Aβ toxicity.	(Haghani et al., 2012)
Synthetic cannabinoid: WIN55,212–2, HU−210 JWH−133 AM251: SR141716 (Rimonabant)	**In vivo/ in vitro:** - Wistar Rat injection models -molecules was co-administered with peptides via intracerebroventricular injection (10 μg in 10 μl of 20 % DMSO/80 % saline per day). - cultured microglial cells, and rat cortical cocultures. - Cannabinoids and βA peptides were added to cultures	**Neuroinflammation:** ⬆️ CB1 and CB2 receptors are present in senile plaques of AD patients and linked to microglial activation. ⬆️ AD brains show reduced G-protein coupling and CB1 receptor expression, with increased nitration of CB1 and CB2 proteins. **Neuroprotection:** ⬆️ WIN55,212–2 and other synthetic cannabinoids prevent Aβ-induced microglial activation, cognitive impairment, and neuronal loss in rats. ⬆️ Cannabinoids also block Aβ-induced microglial activation and **neurotoxicity in vitro.** Cannabinoid Receptor Modulation: Cannabinoid receptors play a key role in AD pathology, and cannabinoids may help prevent neurodegenerative processes.	(Ramírez et al., 2005)
Synthetic cannabinoids: -WIN 55, −212–2 -JWH−133	**In vivo:** - Transgenic amyloid precursor protein (APP) mice (AD model) - Oral administration via drinking water (0.2 mg/kg/day for 4 months)	**Cognitive Function**: ⬆️ JWH−133 normalized novel object recognition deficits in APP mice; WIN 55,212–2 was ineffective. No cognitive changes were observed in wild-type mice. **Brain Glucose Metabolism**: ⬆️ JWH−133 counteracted decreased 18FDG uptake in the hippocampus and cortical regions in APP mice. **Neuroinflammation**: ⬆️ JWH−133 normalized the increased density of Iba1-positive microglia in APP mice. ⬇️ Both cannabinoids reduced elevated COX−2 protein levels and TNF-α mRNA expression. **Amyloid-β Levels**: ⬇️ Both cannabinoids significantly reduced increased cortical β-amyloid (Aβ) levels in APP mice. **Aβ Clearance**: ⬆️ Both cannabinoids enhanced Aβ transport across choroid plexus cells in vitro.	(Martín-Moreno et al., 2012)
-Phytocannabinoids (Δ9-THC and CBD) -Synthetic cannabinoids receptor agonists: (ACEA and JWH−015) - Endocannabinoids:(AEA)	**In vitro** - Rat phaeochromocytoma cells (Ordway PC12). -SH-SY5Y human neuroblastoma cell -incubated for 48 h	**Neuroprotection:** ⬆️ Cannabidiol improved cell viability against tert-butyl hydroperoxide-induced oxidative stress but not against hydrogen peroxide. ⬆️ Anandamide inhibited β-amyloid (Aβ)-induced neurotoxicity in PC12 cells, independent of CB1 or CB2 receptor activation. ⬇️ CB1 agonist ACEA and CB2 agonist JWH−015 did not protect against Aβ or oxidative stress. **Aβ Aggregation and Fibrils:** ⬇️ None of the cannabinoids disrupted preformed Aβ fibrils and aggregates. **Mechanism of Action:** Anandamide protects against Aβ via a receptor-independent pathway. Cannabidiol's protection against oxidative stress does not extend to Aβ exposure.	(B. S. Harvey et al., 2012)
-Phytocannabinoids: (CBD) and (THC) -Endocannabinoids: 2-Arachidonoyl glycerol (2-AG) -Anandamide -Synthetic ECS modulator: ACEA; JWH−015 GPR18/GPR55	**In vitro**: - Neuroblastoma (SH-SY5Y) cells exposed to β amyloid (Aβ1–42). - Microglial (BV−2) cells activated with lipopolysaccharide (LPS). -incubation for 24 h	**Neuroprotection**: ⬆️ 2-AG and CBD directly protected SH-SY5Y cells from Aβ-induced toxicity. ⬆️ JWH−015, Δ9-THC, CBD, Abn-CBD, and O−1602 protected cells from LPS-activated BV−2 conditioned media. **Aβ Toxicity**: ⬇️ Aβ1–42 reduced SH-SY5Y cell viability but did not significantly activate BV−2 cells. **Aβ Aggregation and Morphology**: ⬆️ CB ligands altered Aβ fibril morphology, but this did not clearly correlate with neuroprotection. **Mechanism of Action**: Findings suggest CB ligands protect both microglial and neuronal cells.	(Janefjord et al., 2014)
-Endocannabinoids: Anandamide and noladin ether **(2-AGE)** -antagonist of the cannabinoid type 1 (CB1) receptor: **AM251** -endocannabinoid reuptake inhibitor: **AM404** -selective antagonist of the cannabinoid type 2 (CB2) receptor: **AM630**	**In vitro:** -Differentiated human teratocarcinoma cell line (Ntera 2/cl-D1 neurons) - Incubation in Anandamide and Noladin ether (2–1000 nM)	**Neuroprotection**: ⬆️ Anandamide and noladin ether reduce Aβ toxicity in Ntera 2/cl-D1 neurons. **Cannabinoid Receptor Modulation**: ⬇️ Protection is blocked by the CB1 antagonist AM251. **MAPK Pathway**: ⬇️ Inhibition of the MAPK pathway with PD98059 also prevents cannabinoid protection. **Potential Mechanism**: Cannabinoids and corticotrophin-releasing hormone may use the MAPK pathway to counteract Aβ-induced neurodegeneration.	(Milton, 2002)
Phytocannabinoids: cannabidiol (CBD). Synthetic mixed CB(1)/CB(2) agonist:WIN 55,212–2 (WIN). CB(2)-selective agonists:JWH−133 (JWH) and HU−308 (HU)	**In vitro** -Primary Rat Microglial Cultures prepared from neonatal rat cortex -BV−2 Microglial Cells. -N13 Microglial Cells - Concentration-dependent treatment of cannabinoids **In vivo** -Aβ-Injected C57/Bl6 Mice. - Subchronic intraperitoneal treatment with the cannabinoids (20 mg/kg CBD; 0.5 mg/kg HU−308, JWH, and WIN)	**Neuroinflammation**: ⬇️ CB2-selective agonist reduced ATP-induced intracellular calcium increases in microglial cells. ⬇️ All cannabinoids decreased lipopolysaccharide-induced nitrite generation. **Microglial Function and Migration**: ⬆️ CBD modulated microglial cell function both in vitro and in vivo. ⬆️ CBD demonstrated beneficial effects in an AD model. ⬆️ CBD promoted microglial migration, possibly aiding in Aβ peptide removal, involving cannabinoid and adenosine A(2 A) receptors. ⬆️ CBD and WIN decreased ATP-induced intracellular calcium increase. ⬇️ HU had no effect on intracellular calcium. ⬇️ CBD- and WIN-induced microglial migration was blocked by CB(1) and/or CB(2) antagonists. ⬇️ JWH and HU-induced migration was blocked by CB(2) antagonist only. **Cognitive Function**: ⬆️ CBD and WIN prevented learning deficits and cytokine expression in β-amyloid-injected mice. **Therapeutic Potential**: ⬆️ CBD shows potential as a non-psychoactive therapeutic for AD.	(Martín-Moreno et al., 2011)

Both natural and synthetic cannabinoids exhibit significant therapeutic potential in addressing AD, with effects on neuroinflammation, cognitive function, and neuroprotection.

**Natural cannabinoids**, such as CBD, CBG, and Δ9-THC, have been shown to reduce neuroinflammation and oxidative stress, enhance cognitive functions, and improve synaptic plasticity. They exert anti-apoptotic effects and modulate the ECS, thereby protecting neuronal cells and mitigating Aβ toxicity. Chronic low-dose Δ9-THC administration has been linked to cognitive improvement in preclinical studies, while CBD demonstrates anxiolytic properties and reduces behavioral comorbidities associated with Alzheimer's. These findings highlight the broad therapeutic potential of natural cannabinoids in targeting various aspects of Alzheimer's pathology.

**Synthetic cannabinoids**, including compounds like MDA7, JWH-133, ACEA, and WIN55,212–2, also show substantial promise. They have been reported to improve memory, reduce Aβ levels, normalize neuroinflammatory markers, and offer neuroprotection. Clinical studies with nabilone and dronabinol reveal their effectiveness in alleviating behavioral symptoms such as agitation and disturbed behavior, alongside their anti-inflammatory benefits. In vitro studies further support their role in protecting against Aβ-induced toxicity and microglial activation. Overall, synthetic cannabinoids are emerging as effective agents for managing Alzheimer's, with significant benefits in neuroprotection and inflammation control.

#### 1.2.2 Non-Cannabinoids effect on AD

In the second part of our classification, we shifted our focus to non-cannabinoid compounds derived from cannabis. These compounds have historically remained in the background, overshadowed by the attention given to the ECS. However, their potential therapeutic roles are gaining recognition (Table 4).

**Table 4:** Summary of Studies Investigating the Effects of Non-Cannabinoid Extracts from Cannabis on AD: Experimental Models and Outcomes.

**Non-Cannabinoids**			
Types of Compounds	Experimental Substrate and method of administration	Study Results	References
Terpenes: Terpenes (α-pinene, β-pinene, terpineol, terpinolene, friedelin	**In vivo** -PC12 neuronal cell line exposed to amyloid β (Aβ1–42) protein and oxidative stress induced by tert-butyl hydroperoxide (t-BHP) -Incubation with terpenes (1–200 μM for 24 hr	**Neuroprotection**: ⬆️ Significant neuroprotection observed with α-pinene and β-pinene against amyloid β (Aβ) exposure. **Aβ Aggregation and Fibril Formation**: ⬆️ α-pinene, β-pinene, terpineol, terpinolene, and friedelin reduced Aβ1–42 fibril and aggregate density.	(Laws and Smid, 2024)
Terpenes: (α-bisabolol, myrcene, β-caryophyllene)	**In vitro** -Undifferentiated and differentiated NSC−34 motorneuronal-like cells exposed to amyloid β (Aβ1–42) and tert-butyl hydroperoxide (t-BHP) - Incubation with terpenes (1–1000 μM for 48 hr)	**Neuroprotection**: ⬆️ α-bisabolol provided significant neuroprotective effects against amyloid β (Aβ) exposure and a modest increase in cell viability against oxidative stress. ⬆️ β-caryophyllene showed a small but significant measure of protection against Aβ neurotoxicity. **Aβ Aggregation and Fibril Formation**: ⬆️ Anti-aggregatory effects were observed but not directly correlated with neuroprotective efficacy.	(Laws III et al., 2022)
Terpenes (e.g., 10 different terpenes from *Cannabis sativa*)	**In vitro** Adult rat dorsal root ganglion (DRG) neurons cultured with neurotrophic factors (NGF and GDNF). - Terpenes were administered to DRG neurons for 5 minutes before capsaicin exposure, followed by calcium imaging to monitor neuronal responses.	**Calcium Influx and Neuronal Sensitivity**: ⬇️ Terpenes inhibited capsaicin-evoked calcium influx in DRG neurons, with delayed responses due to calcium release from the endoplasmic reticulum. This inhibition was reversible. **Mechanism of Action**: ⬆️ The inhibition was mediated by Na+ /K+ ATPase activation, not CB1 or CB2 receptor pathways. **Neuronal Co-expression**: ⬆️ Immunofluorescence showed TRPV1 co-expression with Na+ /K+ ATPase in most neurons, suggesting a mechanism for terpene action in modulating neuronal hypersensitivity.	(Anand et al., 2023)
Terpenes: (β-caryophyllene, α-pinene, including (+) and (-) enantiomers)	**In vivo** - Zebrafish - Acute administration in water at different concentrations: -β-caryophyllene: 0.02 %, 0.2 %, 2.0 %, 4 % - α-pinene: 0.01 %, 0.02 %, 0.1 % (including (+) and (-) enantiomers)	**Behavioral Effects**: ⬆️ β-caryophyllene exhibited sedative effects at the highest dose (4 %). ⬆️ α-pinene demonstrated dose-dependent effects on anxiety-like behavior and locomotor activity. The enantiomers (+) and (-) had specific effects on anxiety measures, swimming velocity, and immobility in the open field test, with a minor effect at 0.1 % concentration.	(Johnson et al., 2022)
Flavonoids and polyphenol derivatives (e.g., luteolin, ferulic acid	**In-silico** (computational) analysis. -Virtual screening, molecular docking, and molecular dynamics (MD) simulation.	**Neuroprotective Properties and Binding Affinity**: ⬆️ Luteolin and Ferulic Acid exhibited the highest binding energy (−10.5 kcal/mol) with the target protein PTPRZ, demonstrating neuroprotective properties. **Therapeutic Potential**: ⬆️ The study suggests that these compounds have significant potential as pathway inhibitors in glioblastoma multiforme (GBM) and molecular modulators in AD, contributing to more integrative therapeutic approaches for AD.	(Suhail et al., 2023)
Flavonoids: Prenylated flavonoids (Cannflavin A)	**In vitro** -PC12 cells exposed to amyloid β (Aβ1–42) and tert-butyl hydroperoxide (t-BHP) - Incubation with flavonoids (1–200 μM for 48 hr)	**Neuroprotection**: ⬆️ Cannflavin A demonstrated a hormetic effect, increasing cell viability by up to 40 % at lower concentrations (1–10 μM) and exhibiting neurotoxicity at higher concentrations (>10–100 μM). At 10 μM, cannflavin A inhibited Aβ1–42-induced neurotoxicity, reducing aggregate adherence and neurite loss. **Aβ Aggregation and Fibril Formation**: ⬆️ Cannflavin A directly inhibited Aβ1–42 fibril and aggregate density, with reduced ThT fluorescence and altered Aβ fibril morphology observed through electron microscopy.	(Eggers et al., 2019)

Non-cannabinoid compounds, such as terpenes and flavonoids, show significant promise in addressing AD features, particularly in neuroprotection and reducing Aβ aggregation. Terpenes like α-pinene, β-pinene, and β-caryophyllene have demonstrated neuroprotective effects against Aβ-induced toxicity and oxidative stress, as well as the ability to reduce Aβ fibril formation. Flavonoids, such as cannflavin A, exhibit similar protective effects, particularly at lower concentrations, where they enhance cell viability and inhibit Aβ aggregation. Additionally, some terpenes modulate neuronal hypersensitivity and calcium influx, suggesting a broader mechanism of action that could be beneficial in neurodegenerative conditions. Overall, these natural compounds represent a potential multi-target approach for mitigating AD pathology.

#### 1.2.3 Direct Cannabis extracts

In this section we highlight cannabis extracts that contain both cannabinoid and non-cannabinoid compounds. Although the number of studies on these extracts is relatively small, there is growing evidence of the unique effects of mixed compounds, underscoring the importance of the entourage effect (Table 5).

**Table 5:** Summary of Studies Investigating the Effects of Whole-Plant Cannabis Extracts Containing Both Cannabinoid and Non-Cannabinoid Compounds on AD: Experimental Models and Outcomes.

**Direct Cannabis Extracts:** **Full-Spectrum Extracts:** Cannabinoids and non Cannabinoids				
Types of Compounds	Extraction Method	Experimental Substrate and method of administration	Study Results	References
Marijuana, containing exo-cannabinoids.		**In vivo** Male rats with 6-hydroxy dopamine (6-OHDA)-induced cognitive impairments (in vivo model). - 6-OHDA injected into the substantia nigra to induce cognitive impairments. -Marijuana intraperitoneal injection (60 mg/kg) for 28 days, starting one week after the 6-OHDA injection.	**Cognitive Function**: ⬆️ Marijuana improved spatial learning and memory impairments caused by 6-OHDA in both the MWM and novel object recognition tests. **Dopamine Receptor Expression**: ⬆️ In 6-OHDA-treated animals, marijuana increased D1 mRNA levels but did not affect D2 mRNA levels. **Cannabinoid Receptor Expression**: ⬆️ Marijuana decreased CB1 mRNA and increased CB2 mRNA levels, reversing the effects of 6-OHDA which had increased CB1 and decreased CB2 mRNA levels. **Potential Therapeutic Implications**: ⬆️ Findings suggest marijuana may positively affect learning and memory disorders by altering dopamine and cannabinoid receptor expression, relevant to conditions like Parkinson’s disease.	(Haghparast et al., 2023)
Solvent extract of aerial part of cannabis	Various solvents for extraction of Aerial parts of *Cannabis sativa L* including: hexane, dichloromethane, dichloromethane (1:1), and methanol*.*	**In vitro** pre-adipocytes cell lines	**Cholinesterase Inhibition**: ⬆️ Hexane and dichloromethane extracts exhibited better inhibitory potential against cholinesterase activity. **Cytotoxicity**: No cytotoxic effects were observed in normal Vero and pre-adipocyte cell lines after 24- and 48-hour exposures. **Cannabidiol Concentrations**: ⬆️ Cannabidiol concentrations were higher in the hexane and dichloromethane extracts compared to other solvent extracts. **Potential AD Impact**: ⬆️ The extracts may have potential effects on AD by inhibiting cholinesterase and β-secretase enzyme activities, without cytotoxic effects.	(Mooko et al., 2022)
*Cannabis sativa* extract	mixture of two cannabis extracts 1:1 **-extract 1:** Δ9-THC BDS: Containing 9 % Δ9-THC, 0.9 % cannabigerol, 0.9 % cannabichromene, and 1.9 % other phytocannabinoids **-extract 2:** CBD BDS: Containing 64.8 % CBD, 2.3 % Δ9-THC, 1.1 % cannabigerol, 3.0 % cannabichromene, and 1.5 % other phytocannabinoids.	**In vivo** - A mouse strain was generated by crossing APP/PS1 transgenic mice with CB2 knockout mice. -intra-peritoneally injection of mixture (0.75 mg/kg each) for 5 weeks,	**Labeled Results:** **Cognitive Function**: ⬆️ The combination of Δ9-THC and CBD extracts reduced memory and learning impairments in APP/PS1 mice. **Amyloid-β Plaque Content**: ⬇️ Increased Aβ42 plaque content was observed, regardless of CB2 receptor presence. **Astroglial Reactivity**: ⬇️ Decreased astroglial reactivity was observed, regardless of CB2 receptor presence. **CB2 Receptor Deficiency**: ⬆️ CB2 receptor deficiency exacerbated cortical Aβ deposition and increased soluble Aβ40 levels. ⬎ No effect on tau phosphorylation or microglial reactivity. ⬆️ The positive cognitive effects of the cannabis-based treatment were not affected by CB2 receptor deficiency.	(Aso, Andrés-Benito, Carmona, et al., 2016)
Phytocannabinoids (CBD and THC) and cannabis extracts.	Supercritical carbon dioxide extraction (for cannabis extracts E1, E2, E3, E7, E8).	**In vitro** - Differentiated human neuronal SY-SH5Y cells (in vitro model). -Treatment of cells with CBD, THC, and cannabis extracts at various concentrations; oxidative stress induced by hydrogen peroxide and amyloid-β1–42 in the presence of Cu(II).	**Oxidative Stress:** ⬆️THC showed high potency in combating oxidative stress in both in vitro models. ⬇️ CBD did not exhibit significant antioxidant activity.	(Raja et al., 2020)
The *Cannabis sativa* extract, rich in THCV (THCV-BDS)	Non-psychotropic phytocannabinoid Δ9-tetrahydrocannabivarin (THCV) and a *Cannabis sativa* extract with high THCV content (64.8 %).	**In vitro** studies using lipopolysaccharide (LPS)-stimulated murine peritoneal macrophages	**Receptor Affinity:** THCV-BDS and THCV: Showed similar affinity for CB1 and CB2 receptors in binding assays. **Nitrite Production:** ⬇️ Both THCV-BDS and THCV inhibited nitrite production in LPS-stimulated macrophages via CB2 receptor activation, but not through CB1 receptor pathways. **Enzyme and Cytokine Expression:** ⬇️ THCV down-regulated iNOS, COX−2, and IL−1β protein over-expression induced by LPS. **Receptor Regulation:** ⬇️ THCV counteracted LPS-induced up-regulation of CB1 receptors without affecting CB2, TRPV2, or TRPV4 mRNA expression. **Channel Expression:** ⬇️ TRPA1, TRPV1, TRPV3, and TRPM8 channels were poorly expressed or undetectable in both unstimulated and LPS-challenged macrophages.	([Bibr bib24][Bibr bib133])
*Cannabis sativa L.* extract	Ethanol extraction of dried *Cannabis* flowers, with no purification.	***In vivo*** Carageenan- and formalin-induced paw edema rat models. ***In vitro*** LPS-activated rat monocytes	**Anti-Inflammatory:** ⬇️ Suppression of TNF-α: COE significantly suppressed TNF-α release in LPS-stimulated rat monocytes. ⬇️ Inhibition of COX−2 and i-NOS: COE inhibited LPS-induced COX−2 and i-NOS expression. ⬇️ MAPK Pathway Inhibition: COE blocked the phosphorylation of MAPKs (ERK, JNK, p38). ⬇️ Reduction of Paw Edema: COE significantly inhibited paw edema in rat models. ⬇️ Histopathological Findings: Reduction in inflammation and edema was observed in the chronic paw edema model.	(Shebaby et al., 2021)

Cannabis extracts have demonstrated a range of significant effects on AD-related features and neuroinflammatory responses. In in vivo studies, marijuana improved cognitive function in 6-OHDA-induced rat models by enhancing spatial learning and memory while also modulating dopamine and cannabinoid receptor expression. Additionally, extracts with a combination of THC and CBD showed positive effects on memory and reduced Aβ plaque content in APP/PS1 transgenic mice, independent of CB2 receptor presence.

In vitro studies highlighted the potential of cannabis extracts in inhibiting cholinesterase activity without cytotoxicity, suggesting a protective role against AD. THC, in particular, exhibited strong antioxidant properties, whereas CBD showed limited efficacy in combating oxidative stress.

In studies involving LPS-stimulated murine macrophages, THCV and THCV-BDS demonstrated anti-inflammatory effects by inhibiting nitrite production and down-regulating pro-inflammatory enzymes and cytokines, primarily through CB2 receptor pathways. COE extracts further supported anti-inflammatory outcomes by suppressing TNF-α, inhibiting COX-2 and iNOS expression, and reducing paw edema and inflammation in rat models.

Overall, cannabis extracts show a multifaceted potential in addressing cognitive impairments, neuroprotection, and inflammation, making them promising candidates for AD therapy.

## 2 Conclusion

Cannabis, encompassing both natural cannabinoids, synthetic cannabinoids, and non-cannabinoid compounds, represents a multifaceted and promising avenue for therapeutic intervention in AD. The therapeutic potential of cannabinoids such as CBD, Δ9-THC, and synthetic counterparts like MDA7 and JWH-133 has been supported by evidence demonstrating their neuroprotective properties, ability to reduce neuroinflammation, enhance cognitive function, and alleviate behavioral symptoms.

Additionally, non-cannabinoid compounds derived from cannabis, including terpenes and flavonoids, contribute significantly to the overall therapeutic profile. These compounds may exert complementary effects and influence the efficacy of cannabinoid-based treatments, highlighting the importance of understanding the "entourage effect"—the synergistic interaction between cannabinoids and non-cannabinoid compounds.

However, while the therapeutic promise is substantial, caution is warranted. The complexity of cannabis chemistry and its interactions with the ECS necessitates careful consideration of preparation methods, dosage, and the specific molecular targets involved. Rigorous research and clinical trials are essential to elucidate how cannabinoids and non-cannabinoid compounds interact within the human body, ensuring that therapeutic applications are both effective and safe. Continued investigation into the diverse effects of cannabis extracts and isolated compounds, as well as their interactions, will be crucial for developing well-defined therapeutic strategies and optimizing their potential benefits in AD management.

## CRediT authorship contribution statement

**Abdelhalem Mesfioui:** Writing – review and editing, Validation, Supervision, Conceptualization. **Hanane Doumar:** Writing – original draft, Methodology, Investigation, Conceptualization. **Hicham El Mostafi:** Writing – review & editing, Methodology, Formal analysis, Conceptualization. **Aboubaker Elhessni:** Writing – review & editing, Supervision, Conceptualization. **Mohamed Ebn Touhami:** Writing – review & editing, Project administration.

## Declaration of Competing Interest

There are no conflicts of interest among all authors.
